# Aryl hydrocarbon receptor is involved in DNA damage repair after liver injury with diethylnitrosamine in mice

**DOI:** 10.1186/s43556-026-00596-0

**Published:** 2026-09-29

**Authors:** Beatriz Marín-Díaz, Francisco Javier González-Rico, Laura Ordóñez-Mata, Ángel C. Román, Jaime M. Merino, Pedro M. Fernández-Salguero

**Affiliations:** 1 Departamento de Bioquímica y Biología Molecular y Genética, Facultad de Ciencias, Universidad de Extremadura, Badajoz, 06006 Spain; 2 Instituto Universitario de Investigación Biosanitaria de Extremadura (INUBE), Campus de Badajoz, Avenida de La Investigación S/N, Badajoz, 06006 Spain

**Keywords:** DNA damage repair, AhR, Liver injury, DEN, Cell proliferation, Tissue regeneration

## Abstract

Aryl hydrocarbon receptor (AhR) is a transcription factor traditionally involved in toxicological and carcinogenic cellular processes linked to xenobiotics. In recent decades, a new physiological role has emerged, in which AhR has been shown to be a key regulator of different processes such as the cell cycle, cell proliferation, apoptosis, pluripotency, and stemness. Recent studies have suggested a possible role for AhR in the DNA damage response (DDR) and repair mechanisms. To assess this point, in this work we used diethylnitrosamine (DEN) to induce liver injury and DNA damage in wild type (WT) and AhR knockout (KO) mice. We analysed the protein expression levels of several DDR (H2AX, 53BP1, and BRCA1) and cell proliferation (Ki67, AFP, and PCNA) markers. Therefore, we demonstrate that following DNA damage, the absence of AhR results in a shift towards apoptosis and compensatory proliferation mechanisms, accompanied by reduced repair fidelity of DNA damage compared with than in WT mice, compromising genomic stability. We also mapped the interactome profile of AhR, identifying new partners involved in both repair and proliferation processes. In conclusion, our results highlight the relevant role of AhR in cell proliferation and DNA repair processes, which can be key in different pathologies, such as cancer.

**Supplementary Information:**

The online version contains supplementary material available at https://doi.org/10.1186/s43556-026-00596-0.

## Introduction

The transcription factor aryl hydrocarbon/dioxin receptor (AhR) is structurally included within the class I basic helix-loop-helix transcriptional regulators [1–5]. This protein has been traditionally studied for its roles in toxicology and carcinogenesis in response to dioxins such as 2,3,7,8-tetrachlorodibenzo-p-dioxin (TCDD) [6, 7]. However, subsequent studies have revealed the importance of AhR in different physiological and homeostatic processes, such as signalling pathways [8] regulating the cell cycle, cell proliferation, apoptosis [9], cell morphology, cell adhesion and migration [10], angiogenesis [11], differentiation [12], chromatin dynamics [13, 14], pluripotency, and stemness [15–17].

Most mammalian tissues present limited cell renewal under normal homeostatic conditions. However, some organs such as the lung or liver, exhibit a rapid regenerative response when exposed to toxic or harmful agents. In fact, the role of AhR in tissue regeneration has been previously described in murine liver and lung [18–21]. Thus, these organs can recover their normal structure and function by activating undifferentiated precursor cells after damage caused by chemicals [20].

Interestingly, some authors have described the role of AhR in DNA damage repair [22–25]. Transcriptomic analyses have demonstrated that AhR is an essential transcription factor for coordinating the DNA damage response (DDR) following exposure to chemical carcinogens, such as benzo(a)pyrene and aflatoxin B1 [22]. In addition to chemical ligands, AhR responds to physical stimuli in a context-dependent manner. Upon exposure to ionizing radiation, AhR translocates to the nucleus to upregulate DNA repair and cell survival [23], a protective effect similar to that observed under oxidative stress, where it enhances the DDR and suppresses tumour progression [24]. Conversely, in response to ultraviolet radiation, AhR plays a detrimental role by repressing the nucleotide excision repair (NER) pathway and blocking apoptosis, thereby promoting cutaneous carcinogenesis [25]. However, its role in tissue regeneration after DNA damage in the liver remains to be elucidated.

Diethylnitrosamine (DEN) is widely used to induce liver injury and tumorigenesis in experimental animal models [20, 26–28]. Following DEN absorption by the liver, it is metabolized by cytochrome P450 enzymes, such as CYP2E1, which convert it into the more reactive compound hydroxynitrosamine. It then rapidly breaks down into a highly reactive ethyldiazonium ion, which covalently binds to DNA bases, mainly guanine, forming adducts that alter DNA structure and can cause mutations, disrupt transcription, and increase the risk of cancer over the time. DEN may result in the production of DNA adducts in any tissue with appropriate activation of P450 isozymes [29, 30]. It has been reported that exposure to DEN for short periods and at high concentrations induces acute hepatotoxicity, DNA damage and mutations, and can even result in death in humans [28, 29].

In this work, we studied the role of AhR in tissue regeneration after acute liver injury with DEN and subsequent DNA damage by using wild type and AhR knockout mice as experimental models and analysing the protein network involved in DNA repair and cell cycle progression. [26, 31]. We describe how AhR contributes to maintaining a more robust and accurate DNA repair mechanism via homologous recombination (HR). Interestingly, its absence drives the cell into a more proliferative state, opting for nonhomologous DNA end joining (NHEJ) repair, suggesting a presumable compensation route due to its less efficient reparation mechanism.

## Results

### The absence of AhR increases the proliferation rate and affects the recovery of hepatocellular integrity after acute liver damage

**Fig. 1:**
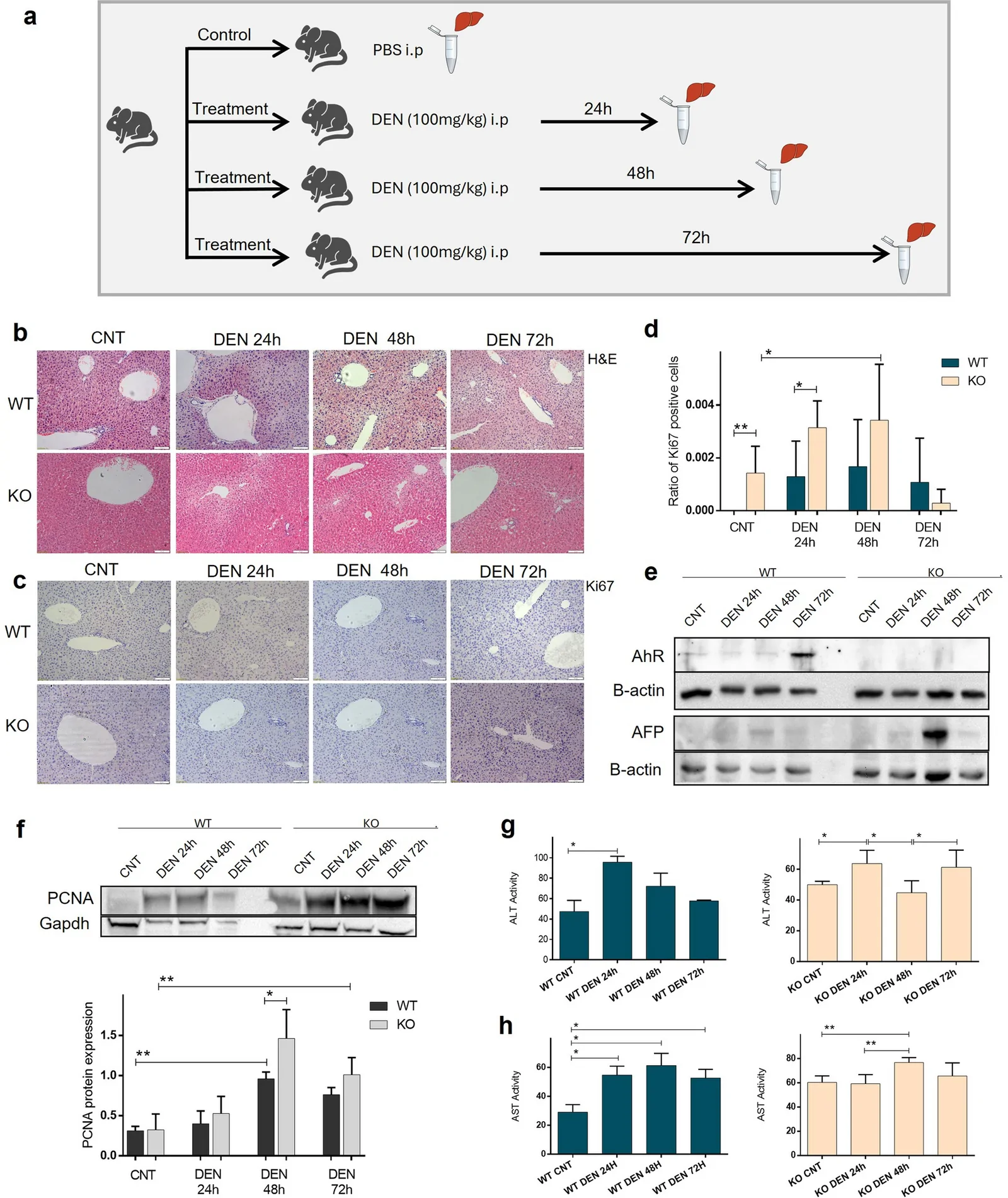
Absence of AhR increases the hepatic proliferation rate and affects the recovery of hepatocellular integrity following liver injury. **a** Schematic diagram of the acute liver injury protocol for DEN treatment.** b** Representative images of haematoxylin and eosin (H&E) stained liver sections from WT and KO mice for the untreated control group (CNT) and 24, 48, and 72 h after DEN treatment. **c** Analysis of Ki67 expression by immunohistochemistry in both genotypes for each experimental condition. **d** Relative cell proliferation was measured as the Ki67-positive cell ratio. A Nikon TE2000U microscope was used to obtain the immunohistochemistry images. Representative scale bars correspond to 100 µm. AhR, AFP (**e**) and PCNA (**f**) protein levels were analysed in liver extracts by immunoblotting. β-actin was used as a loading control. The quantification of PCNA protein levels for each condition is shown below (**f**). Hepatic alanine aminotransferase (**g**) and aspartate aminotransferase (**h**) activity in treated mice. The data are shown as the mean ± SD. * *p* < 0.05, ** *p* < 0.01, *** *p* < 0.001. Groups of 6–8 mice were used for each genotype and time point, and each experiment was repeated (*n* = 2–4)

We first analysed the effect of the absence of AhR on cell proliferation after liver injury induced by DEN. Wild type (WT) and AhR KO mice were treated with DEN for 24, 48 and 72 h (Fig. 1a). As shown in Fig. 1b., H&E staining of liver sections from WT and KO mice revealed mild histopathological differences, with KO animals exhibiting increased apoptosis and focal areas of necrosis compared with those of WT mice. However, Ki67 expression, a marker for cell proliferation, increased at 24 and 48 h after DEN treatment in the KO genotype, followed by a significant decrease at 72 h (Fig. 1c-d). This increase was significantly different from that in the WT genotype (Fig. 1 c-d). Next, we assessed AhR regulation. As expected, AhR expression was undetectable in the KO animals. In contrast, protein AhR levels were significantly increased in WT mice at 72 h after treatment (Fig. 1e). Additional proliferation markers, including AFP and PCNA, were subsequently examined under the same conditions to further define the proliferative phenotype. AFP expression increased at 48 h after DEN treatment in both genotypes, with significantly higher levels in the KO animals than in the WT animals (Fig. 1e). Consistent with the AFP response, PCNA expression (Fig. 1f) was increased at 24–48 h following DEN treatment, with significantly higher levels observed in the KO animals than in the WT animals.

Following the assessment of liver proliferation markers, hepatic damage was addressed by measuring transaminase levels. Thus, alanine aminotransferase (ALT) levels were significantly altered at 24 h in both genotypes (Fig. 1g). Whereas WT mice subsequently recovered to baseline, KO mice sustained significantly elevated ALT levels at 72 h after DEN treatment (Fig. 1g). Interestingly, a distinct pattern was observed for serum AST levels (Fig. 1h), where compared with WT controls, KO mice displayed significantly higher baseline AST levels. After DEN treatment, AST levels in WT mice increased steadily, peaking at 48 h and slightly declining by 72 h. In contrast, the levels in KO mice increased only modestly at 48 h, followed by a slight decrease at 72 h (Fig. 1h). Taken together, these results suggest that the absence of AhR accelerates and improves the hepatic proliferative response, but delays the normalization of transaminase levels following liver injury.

### The absence of AhR exacerbates the apoptotic response after damage

**Fig. 2:**
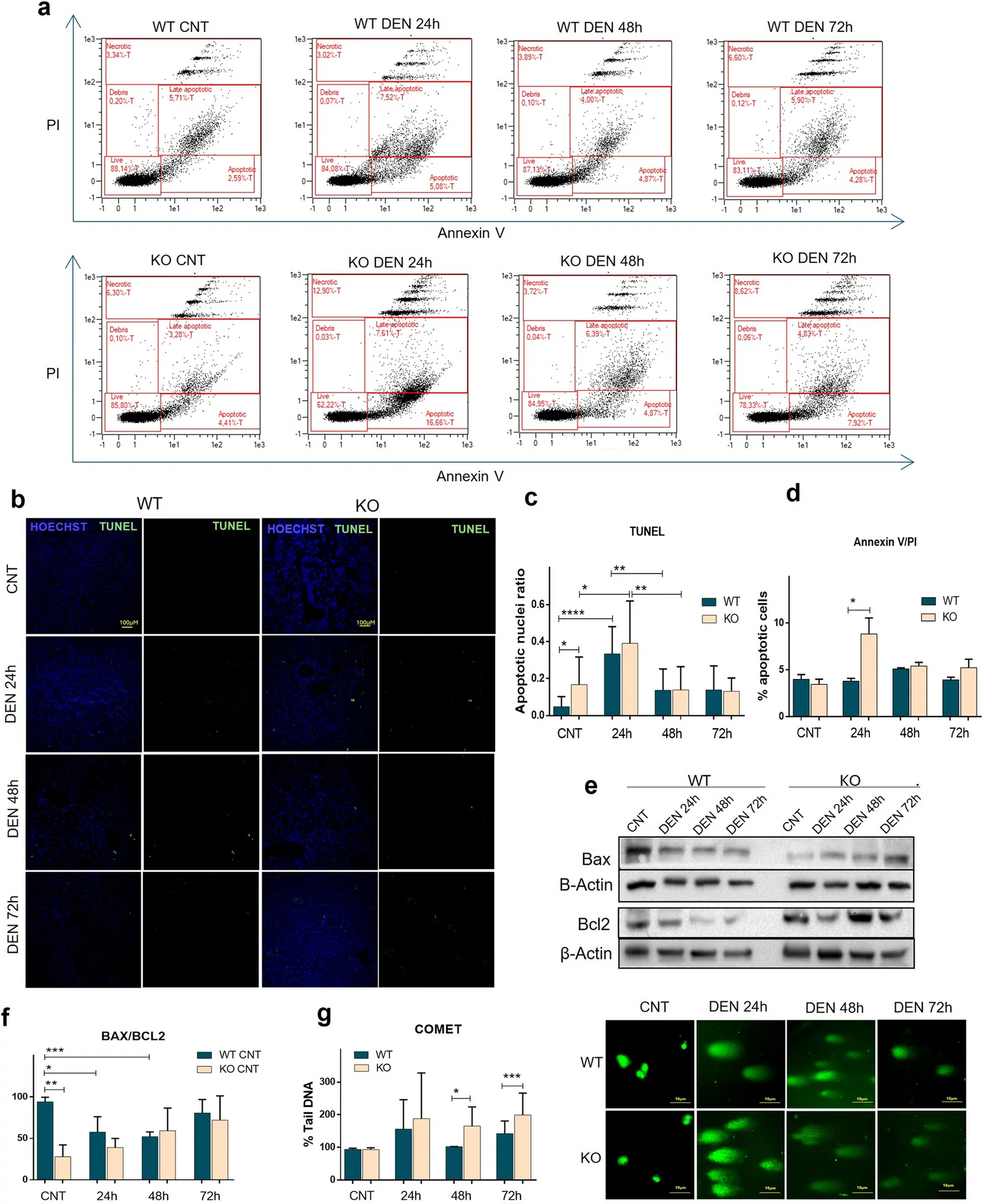
DEN treatment induces apoptosis and DNA damage in both genotypes, with a greater apoptotic response in KO mice. **a** Representative flow cytometric quadrant dot plots of Annexin V-FITC/PI staining. **b** Representative micrographs of apoptotic cells assessed by a TUNEL assay, with Hoechst staining used to label cell nuclei. Representative scale bars are 100 µm. **c** Relative number of apoptotic cells among total nuclei measured as the number of TUNEL-positive nuclei. **d** Relative percentage of apoptotic cells (early + late apoptosis) quantified from the flow cytometry profiles shown in (**a**) panel. **e** BAX, and BCL2, protein levels were analysed in liver extracts by immunoblotting. β-actin was used as a loading control. **f** Densitometric quantification of the BAX/BCL2 protein ratio using loading-control-normalized protein levels. **g** Representative comet assay images and respective tail moment quantification. Representative scale bars are 10 µm. Images from the **b** and **g** panels were captured using an Olympus FV1000 confocal microscope and FV10 software. The data are shown as the mean ± SD. ∗ *p* < 0.05; ∗ ∗ *p* < 0.01; ∗ ∗ ∗ *p* < 0.001. Number of mice (*N* = 3) and replicates of each experiment (*n* = 2–3)

We then addressed cell and DNA integrity after acute damage. The liver damage caused by DEN induced apoptosis, as shown in Fig. 2. Apoptotic cells were quantified by flow cytometry using Annexin V-FITC and propidium iodide (Fig. 2a and d), which revealed an increase in apoptosis 24 h after DEN treatment in the KO mice. To strengthen these findings and expand our evidence of cell death, we subsequently performed a TUNEL assay under the same experimental conditions. Interestingly, increases in TUNEL-positive nuclei were observed in both genotypes (Fig. 2b-c). Additionally, the levels of cleaved Casp3 were analysed for both genotypes, and were similar to the results observed by TUNEL assay, with an increase at 24 h after DEN treatment (Fig. S1). Additionally, the antiapoptotic protein Bcl2 and the proapoptotic protein Bax were measured by immunoblotting across all the experimental conditions (Fig. 2e), and their ratios were analysed (Fig. 2f). Compared with TUNEL, the BAX/BCL2 ratio showed inverse kinetics, indicating a potential compensatory response. To further define the apoptotic response observed, DNA damage was evaluated using the comet assay (Fig. 2g). Treatment with DEN resulted in significant increases in the percentage of DNA in the tail in both genotypes at 24 h. This effect was transient in WT mice, which recovered by 72 h, whereas KO mice exhibited persistent DNA damage (Fig. 2g). These results suggest that, despite having similar levels of DNA damage, the apoptotic response is faster and more pronounced in the KO mice than in the WT mice.

### AhR deficiency leads to a less efficient and less faithful repair mechanism

**Fig. 3:**
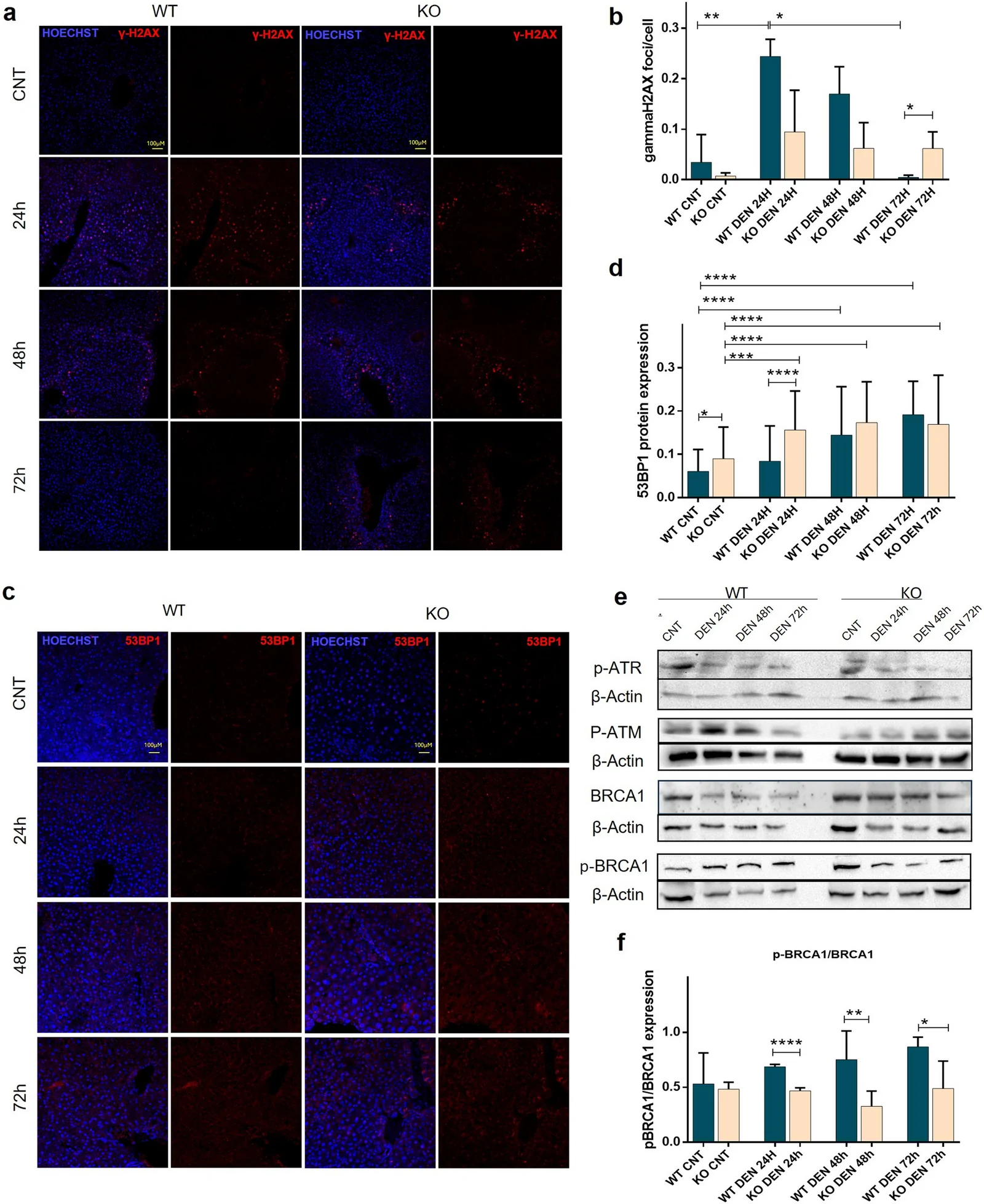
KO mice show defective signalling of damage via gamma-H2AX and an increase in 53BP1 foci. **a** γH2AX foci were analysed by immunofluorescence in liver sections. Hoechst staining was used to label the cell nuclei. The scale bars represent 100 µm. **b** Quantification of the percentages of γH2AX-positive hepatocytes in livers from WT and KO mice. **c** 53BP1 foci were analysed by immunofluorescence in liver sections. Hoechst staining was used to label the cell nuclei. The scale bars represent 100 µm. **d** Quantification of the percentages of 53BP1-positive hepatocytes in livers from WT and KO mice. **e** p-ATR, p-ATM, BRCA1 and p-BRCA1, protein levels were analysed in liver extracts by immunoblotting. β-actin was used as a loading control. **f** Densitometric quantification of the [p-BRCA1]/[BRCA1] protein ratio using loading-control-normalized protein levels. Immunofluorescence images were acquired using an Olympus FV1000 microscope and analysed with FV10 software (Olympus). The data are shown as the mean ± SD. ∗ *p* < 0.05; ∗ ∗ *p* < 0.01; ∗ ∗ ∗ *p* < 0.001. Number of replicates (*N* = 5) and measurements taken from 4–6 fields for each replicate

DEN treatment causes liver injury that can affect DNA integrity [29, 30]. Consistent with our previous observation (Fig. 2g), although DEN exposure initially induced equivalent levels of DNA damage in both genotypes, KO mice exhibited incomplete recovery by 72 h, indicating an impairment in DNA repair efficiency. To assess this point we measured the DNA damage marker γH2AX in both genotypes after DEN treatment. The expression of γH2AX was greater in the WT genotype than in the KO genotype, both at baseline and following DEN treatment. (Fig. 3a and b). After 72 h, the γH2AX labelling was completely cleared in the WT animals, but remained persistent in the KO group (Fig. 3a and b). Additionally, we analysed the expression of the DNA repair nonhomologous end joining (NHEJ) mediator p53-binding protein 1 (53BP1). Our data revealed that the absence of AhR increased both the basal and DEN treatment-induced expression levels of 53BP1 (Fig. 3c and d). Next, we measured the expression of pBRCA1, a DNA damage repair protein and homologous recombination mediator, which was significantly greater in WT animals than in KO animals (Fig. 3e and f). Finally, we analysed the expression of the Ser/Thr kinase ATM and the Ser/Thr kinase ATR, which are recruited by DNA damage to induce cell cycle arrest. In WT mice, p-ATM expression was significantly upregulated at 24 h postinjury, whereas this acute response was absent in genotype KO mice (Fig. 3e). By 72 h, p-ATM levels in WT mice had decreased to near-basal levels. In contrast, compared with WT genotype, the KO mice exhibited higher p-ATM expression levels (Fig. 3e). These results provide evidence that, despite similar levels of DNA damage in WT and KO mice after acute injury, the detection of this damage via γH2AX is impaired in KO mice. Additionally, upon DNA damage, the WT genotype prioritizes the homologous recombination (HR) repair pathway, whereas the KO genotype relies on NHEJ pathway.

### Absence of AhR dysregulates the p53–AKT survival axis

**Fig. 4:**
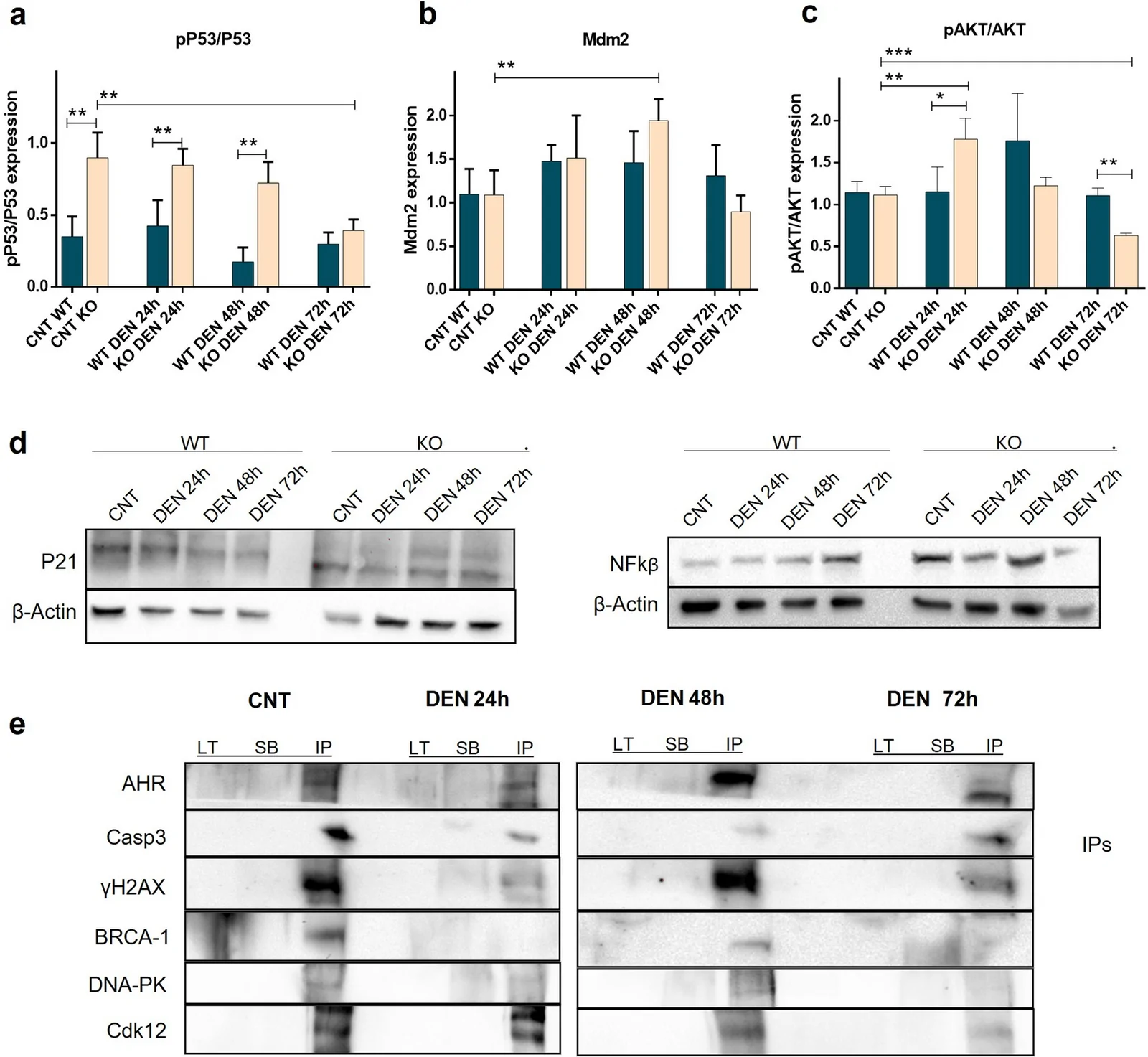
The p53-AKT-mediated response is altered in the absence of AhR, while AhR immunoprecipitates with DNA damage response proteins. **a** Densitometric quantification of the p-p53/p53 and pAKT/AKT (**c**) protein ratios using loading-control-normalized protein levels. **b** Mdm2 protein levels in WT and KO mice after DEN treatment. **d** Analysis of p21 and NFκB protein levels in liver extracts by immunoblotting. β-actin was used as a loading control. **e** AhR immunoprecipitation from the nuclear protein fraction after treatment with DEN. Proteins in complex with AhR were detected with specific antibodies after western blotting. The data are shown as the mean ± SD. ∗ *p* < 0.05; ∗ ∗ *p* < 0.01; ∗ ∗ ∗ *p* < 0.001. Groups of 5–8 mice were used for each genotype and time point, and each experiment was repeated (*n* = 2–4)

Next, we analysed the potential role of AhR in proliferation and cell division in our study model. Interestingly, the p53-AKT pathway was activated differently between the WT and KO genotypes (Fig. 4). Phospho-p53 expression was increased in the KO genotype compared with the WT genotype for each condition, including in control cells without DEN treatment (Fig. 4a). Additionally, the level of phospho-AKT increased after DEN treatment at 24 h in WT mice, whereas this increase was delayed until 48 h in KO mice. Moreover, its expression subsequently decreased at 72 h in both genotypes (Fig. 4c). Similar results were obtained for the expression of Mdm2, a negative p53 regulator, which increased at 48 h after DEN treatment in the KO genotype (Fig. 4b). To complete the analysis of p53 signalling, the expression of p21 and NFκB was analysed by immunoblotting (Fig. 4d). These findings suggest that the p53-AKT mediated response is altered in the absence of AhR.

### AhR interacts with key DDR, proliferation and apoptotic proteins

**Fig. 5:**
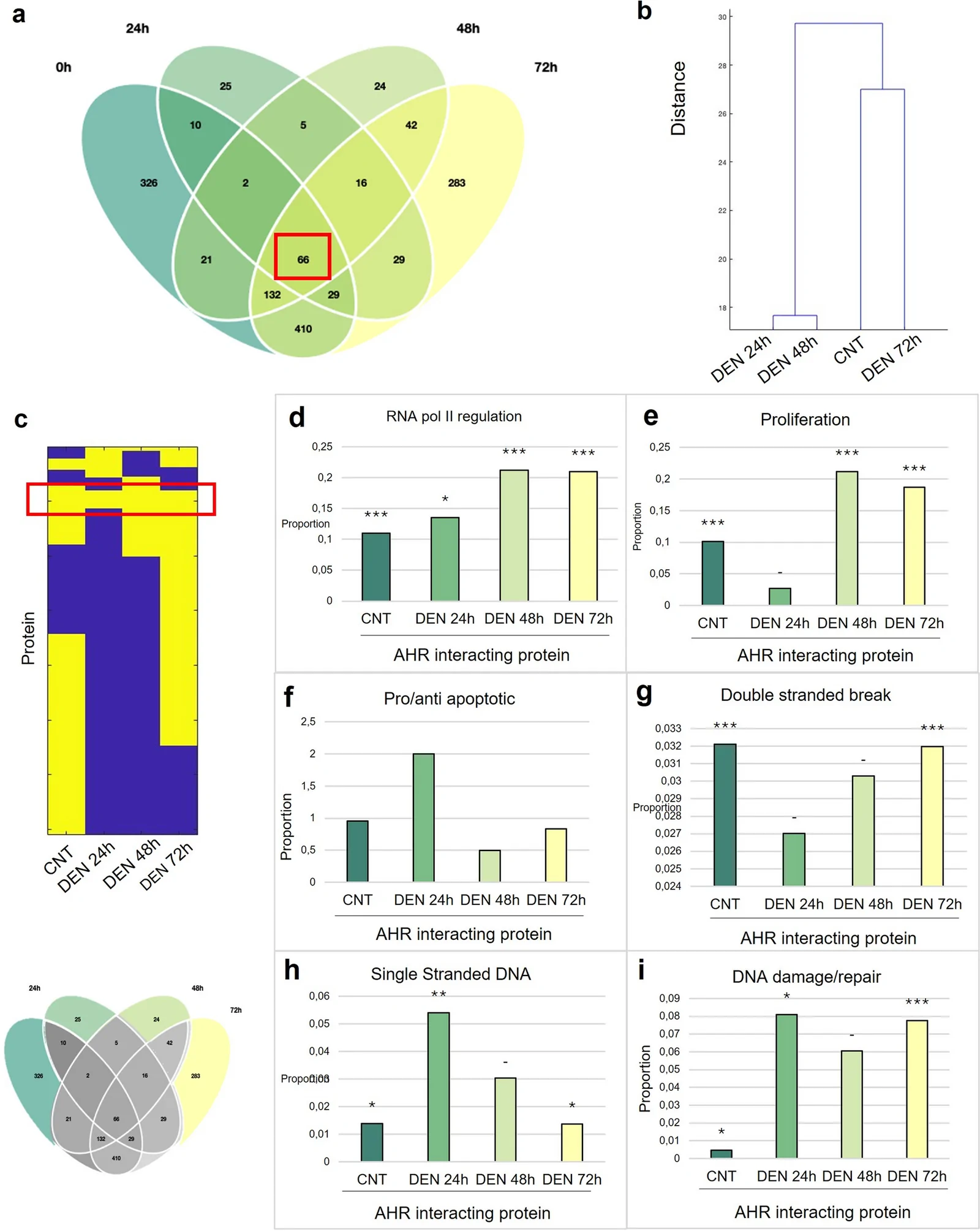
Bioinformatic and Gene Ontology analyses of the AhR interactome in response to DNA damage. **a** Venn diagram illustrating proteins uniquely detected at each time point and those shared between them, highlighting potential common and time-specific AhR-interacting proteins. The red box highlights a cluster of proteins that interact with AhR across all the experimental conditions. **b** Dendrogram showing proteins clustered according to their expression profiles, revealing groups of proteins with similar temporal patterns and potential functional relationships. **c** The heat map colouration indicates the degree of similarity in protein expression profiles across samples: Yellow indicates the presence of interactions, and blue indicates no detected interactions. The red box highlights a cluster of proteins that interact with AhR across all the experimental conditions. Condition-specific AhR-interacting proteins identified by proteomics were further analysed by Gene Ontology enrichment. Representative categories (**d**, **e**, **f**, **g**, **h**, **i**) were selected based on their biological relevance to the study context. Functional annotation was performed using the Database for Annotation, Visualization, and Integrated Discovery (DAVID). The columns indicate the proportion of proteins belonging to each functional category relative to the total number of proteins expressed exclusively under that specific condition. GO enrichment was assessed using Fisher’s exact test. ∗ *p* < 0.05; ∗ ∗ *p* < 0.01; ∗ ∗ ∗ *p* < 0.001

To elucidate the mechanism of action of AhR in DNA damage repair induced by DEN treatment, we performed coimmunoprecipitation experiments to identify the proteins involved in cell death, DNA repair and proliferation bound to AhR. Six proteins, namely, AhR, Casp-3, yH2AX, BRCA1, DNA-PK and CDK12, were coimmunoprecipitated at 24, 48 and 72 h after DEN treatment (Fig. 4e). To expand these findings and achieve a comprehensive view of the interactome, we subsequently performed proteomic analysis of the immunoprecipitated samples, as detailed in the Materials and Methods section. We detected 1420 interactions between AhR and other proteins at different times after treatment (Fig. 5a, and Fig. S2a). We used the matrix of overlapping AhR interactions to quantify the distances between samples. Therefore, we observed that the most similar conditions regarding AhR interaction partners were DEN at 24 h and DEN at 48 h. Interestingly, compared with the other samples, the control and DEN at 72 h were more similar (Fig. 5b and c). These results reflect the modulation of AhR interactions during DEN treatment. We then explored the molecular and functional pathways that were enriched in these AhR interactions during treatment (Fig. 5d-i). We found that statistically significant GO pathways were present at some points during the treatment. RNA pol II activity (related to the role of AhR as a transcriptional regulator) and proliferation were enriched mostly in the CNT, and treated group at 48 and 72 h (Fig. 5d and e), whereas the pro-apoptotic pathway was specifically increased at 24 h (Fig. 5f). Furthermore, proteins involved in the DNA damage response (DDR) and break-marking pathways also exhibited time-dependent binding to AhR following treatment (Fig. 5g-i).

Taken together, these data suggest a model in which protein AhR binds directly to different proteins involved in the DNA damage response, thereby contributing to its proper function in this mechanism.

## Discussion

AhR is known to be involved not only in numerous essential, homeostatic and physiological processes, such as cell function and tissue regeneration, but also in the development of several pathologies, including oncogenesis. Additionally, recent studies address the possible relationship between AhR and DNA damage repair in different tissues and under different genotoxic conditions [32]. Therefore, AhR promotes the response to damage in prostate cancer cells under oxidative stress [24]. Additionally, AhR plays an antiapoptotic role in human epidermal keratinocytes irradiated with UVB [33], influences DNA damage-dependent responses and contributes critically to cutaneous photocarcinogenesis in mice [25].

However, knowledge about how AhR conditions and drives DNA repair in other tissues or organs, such as the liver, is still scarce. Therefore, in this study we attempted to elucidate this new role of the receptor. To do so, we employed AhR-wild type (WT) and AhR-knockout (KO) mice as a model to study, DNA damage caused by acute DEN treatment, and to study the repair responses in both genotypes. Initially, our results revealed that DEN treatment caused tissue damage in both phenotypes, which started to recover after 24 h. Nevertheless, the pattern differed markedly between the two genotypes. Thus, compared with WT animals, KO animals exhibited a compensatory proliferation response mechanism after damage, as indicated by the expression profiles of the PCNA, AFP, and Ki67 markers analysed. In WT animals, the observed increase in AhR expression was paired with a reduction in the proliferation rate (72 h). Furthermore, the levels of the liver stress markers ALT and AST, increased in the KO genotype, indicating that the absence of AhR affects the restoration of hepatocellular integrity following liver injury.

Interestingly, reports from other groups have shown that the activation of the AhR pathway inhibited or slowed the regeneration rate [34]. Moreover, dioxin receptors can regulate the regeneration process by preventing excessive tissue growth [18], whereas its absence promotes the development of hepatocellular carcinoma (HCC) in the long term [20]. These findings were supported by additional TUNEL, annexin V, and cleaved caspase data, which revealed that, although both genotypes underwent apoptosis at 24 h posttreatment, this response was significantly more pronounced in the KO genotype. Additionally, the upregulation of BCL2 in the KO mice could support the compensatory response mechanism mentioned above, helping to counteract the elevated apoptotic activity observed in these animals at 24 h. In support of this hypothesis, the apoptotic levels at 48 h remained similar between the KO and WT mice. Nevertheless, further investigation is needed to elucidate the precise molecular pathways driving this potential feedback mechanism. Taken together, these findings could indicate that AhR contributes to controlling the balance in the regenerative process, preventing uncontrolled proliferation. In the case of KO animals, the absence of this regulator leads to an altered response due to increased apoptosis, massive necrosis, and proliferation.

Comet assay analysis revealed that the magnitude of DNA damage did not differ significantly between genotypes. Nevertheless, WT mice efficiently recovered from this genotoxic insult, whereas the damage remained unresolved at 72 h in KO mice, suggesting that the absence of AhR compromises the efficiency of the DNA repair machinery. To better define the functional involvement of the protein AhR in this context, a panel of DNA damage markers was analysed. Consistent with the previous findings, the results revealed that the expression of the repair machinery recruitment protein following damage (gammaH2AX) was reduced in the KO mice. Such a reduction in the absence of AhR could explain the impaired DNA repair efficiency observed in the KO genotype. In accordance with this result, previous studies have reported that AhR and aryl hydrocarbon receptor nuclear translocator (ARNT) are key to the phosphorylation of H2AX after exposure to aflatoxin B1 (AFB1), a potent liver carcinogen [22], and that AhR is part of H2AX foci following irradiation in ovarian cells, bronchial carcinoma, and keratinocytes [23].

These findings demonstrate that, rather than relying on error detection mechanisms and DNA repair, KO animals preferentially undergo apoptosis and proliferation in response to acute damage. Additionally, analysis of the DNA damage repair markers, p-BRCA1, pATM and 53BP1, revealed clear differences between the two genotypes. These findings suggest that the choice of DNA repair pathway may also be influenced by the presence of AhR, with homologous recombination (HR) predominating under WT conditions. Conversely, in the absence of AhR, nonhomologous end joining (NHEJ) appeared to be the dominant strategy, favouring a less faithful repair and greater early apoptotic activation. Despite this, it has been reported [35] that the suppression of murine AhR effectively restored DNA repair by HR in placental tissues and alleviated spontaneous abortion. The divergence observed in our data may be explained by the fact that AhR displays differential, and sometimes contrasting, behaviour of AhR depending on the organ and cell type studied. Specifically in the placenta, other studies have shown that AhR dynamics are opposite to those found in the liver [36]. Accordingly, our results showed that AhR may be involved in the decision-making process underlying the selection of a repair pathway. Nevertheless, it would be necessary to examine both mechanisms in greater depth to address this possibility. Compared with WT animals, KO animals showed basal overactivation of the tumour suppressor P53. These observations are consistent with previous findings reported by our research group, which revealed that p53 is overexpressed in the livers of adult AhR-/- mice [37]. This observation may again reflect a compensatory response aimed at controlling the tumorigenesis characteristics of KO animals, as well as their increased susceptibility to the development of induced neoplasms [38–43]. Additionally, the negative feedback mechanism mediated by Mdm2 was similar in both genotypes, but was not enough to counteract p53 overactivation in the KO animals. This could be explained by the contribution of AhR to Mdm2-dependent p53 ubiquitination and degradation, as previously described [44–46]. Furthermore, the pAKT/AKT survival pathway also showed similar dynamics in both genotypes, but the time window was delayed. Although the KO model exhibited an early survival response (24 h), this trend was not sustained with a shift towards a phenotype of apoptosis and cell proliferation being observed. In contrast, wild-type mice showed delayed response kinetics, characterized by controlled and homeostatic progression. Consistently, the high NF-κB expression in KO mice reinforces the presence of a sustained proinflammatory state [47, 48]. In contrast, the WT cohort displayed a typical homeostatic adaptation, with levels rising proportionally in response to tissue damage. In fact, our results revealed a physical interaction between AhR and different DNA damage repair and cell death proteins (yH2AX, BRCA1, DNA-PK, CDK12, and Casp3), which is reinforced by the findings reported by other authors [23]. Thus, the data obtained by coimmunoprecipitation and proteomics and gene ontology analysis confirm that AhR binds to a panel of proteins involved in key processes of DNA damage repair, proliferation, and apoptosis regulation, in a condition-dependent manner. Collectively, these data support the hypothesis that AhR actively participates in the repair mechanism.

It is known that KO mice (AhR∆3/∆3) exhibit a phenotype of premature aging and reduced lifespan [49]. Because ageing is characterized by the accumulation of damage to intracellular macromolecules, the deficiency in the DNA damage repair mechanism caused by the lack of AhR we reported could contribute to increased ageing in this phenotype.

Despite providing key insights into the role of AhR in the DNA damage response and tissue regeneration, several limitations of this study should be noted. First, our experimental model based on mice with a constitutive (germline) deletion of AhR strongly supports our hypothesis; however, validation in a liver-specific conditional AhR knockout model would further strengthen our conclusions. Second, all our analyses cover strictly the scope of the first 72 h of response to acute liver injury; hence, it would be interesting to study the mechanism in the long term.

**Fig. 6:**
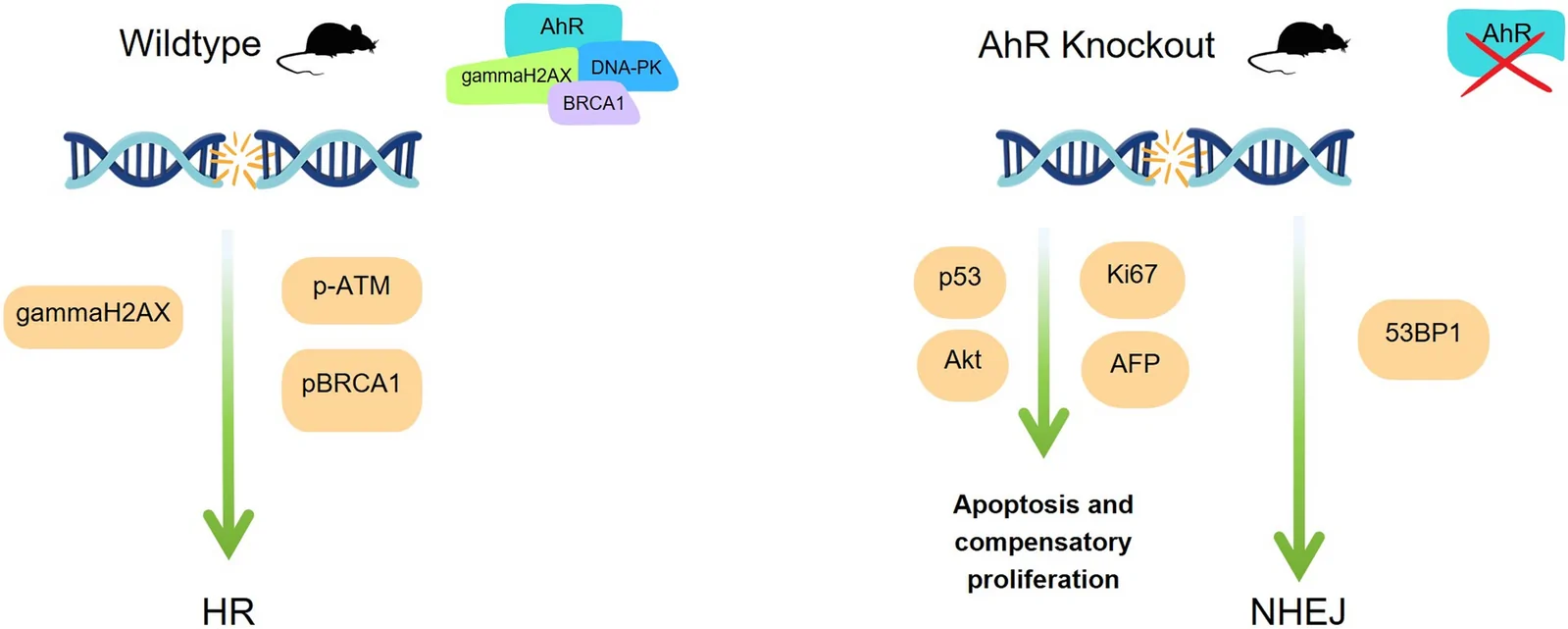
Schematic diagram illustrating the proposed mechanism through which AhR contributes to DNA damage repair after liver injury with DEN

In conclusion, our work describes how AhR is involved in the repair mechanism activated by acute-induced DNA damage, exerting a controlled response and a potential activation of the most faithful repair pathway (HR). Interestingly, the loss of AhR led to less efficient and probably less conservative repair (NHEJ), resulting in a greater apoptosis and cell renewal strategy through a compensatory proliferation mechanism (Fig. 6). Future studies are needed to elucidate how a lack of AhR contributes to increased genomic stress and potential long-term tumorigenesis.

## Materials and methods

### Mice and treatments

Following the procedure reported by [50, 51], AhR +/+ and AhR −/− mice (C57BL6/N × 129/Sv background) were produced through homologous recombination in embryonic stem cells, targeting the deletion of exon 1 of the dioxin receptor gene.

To induce acute liver injury, 10- to 12-week-old mice were injected with 100 mg/kg body weight diethylnitrosamine (DEN) (Sigma N0258) in phosphate-buffered saline (PBS) and sacrificed 24, 48 and 72 h after treatment. The control group received the same volume of phosphate-buffered saline (PBS) as the vehicle control. Animals were provided with ad libitum access to water and standard rodent diet. All animal procedures were conducted in compliance with applicable national and European guidelines for animal welfare (Spanish Royal Decree RD53/2013, amended by RD 1386/2018, and EU Directive 2010/63/EU, amended by Regulation 2019/1010). The institutional Bioethics Committee for Animal Research at the University of Extremadura (Registry 109/2014) and the Junta de Extremadura (EXP-20160506–1) authorized all animal experiments in this study.

### Flow cytometry

Fresh liver tissue was extracted from the mice following euthanasia at the indicated time points after treatment with DEN or, in the case of the control group, after vehicle administration. The tissue was collected in DMEM containing 10% FBS, 100 U/ml penicillin, 100 μg/ml streptomycin, and 2 mM L-glutamine for analysis by flow cytometry. The tissue subsequently underwent mechanical and enzymatic dissociation using a gentleMACS™ Octo Dissociator (Miltenyi Biotec). Following filtration through a 30 µm mesh, the resulting cell suspension was centrifuged at 1,800 rpm for 5 min.

Annexin/propidium iodide staining was used to distinguish healthy cells from cells undergoing early, late, or necrotic apoptosis. The cells were resuspended at an approximate density of (2–5) × 10^5^ cells/mL in 200 µL of 1X Binding Buffer from an Annexin V-FITC Apoptosis Detection Kit (eBioscience, Invitrogen; Ref. BMS500FI-300) and incubated with 5 µL of Annexin V-FITC for 10 min at room temperature. After being washed with 200 µL of buffer, the cells were resuspended in 190 µL of Binding Buffer and stained with 10 µL of propidium iodide (20 µg/mL). The samples were analysed using a MACSQuant® VYB flow cytometer.

### Ki67 and cleaved-Casp3 immunohistochemistry, haematoxylin and eosin staining, and TUNEL Staining

The livers from the mice sacrificed at the indicated times were isolated, and fixed at room temperature in buffered formalin overnight.

The samples were embedded in paraffin and processed for haematoxylin and eosin staining, Ki-67 immunohistochemistry, or a TUNEL assay at the Pathology Service of the Salamanca Cancer Research Center (CIC). Finally, haematoxylin and eosin, c-casp3 and Ki67 sections were observed under a Nikon TE2000U microscope using 4 × (0.10 numeric aperture), 10 × (0.25 numeric aperture), 20 × (0.40 numeric aperture), and 40 × (0.60 numeric aperture) objectives. TUNEL stained sections were visualized using an Olympus FV1000 confocal microscope.

For immunohistochemistry (IHC) quantification, a hybrid method was performed in ImageJ/Fiji. Brightfield images underwent “Colour Deconvolution” to separate the haematoxylin and chromogen signals. Total nuclei were counted automatically from the haematoxylin channel using thresholding, watershed separation, and the 'Analyse Particles' tool. Conversely, marker-positive cells were counted manually based on chromogen staining and morphology by a blinded investigator. The percentage of positive cells was calculated as the number of manually positive cells divided by the total automated nuclear count per field. For TUNEL assay quantification, the analysis was conducted automatically for each independent channel following the same protocol described for immunofluorescence.

### Liver function test

Hepatic ALT activity was measured using a commercial Alanine Transaminase Activity Assay Kit (ab105134, Abcam, UK) according to the manufacturer’s instructions. Liver samples were homogenized in assay buffer, and centrifuged, and the resulting supernatants were used for analysis. ALT activity was determined fluorometrically (Ex/Em = 535/587 nm) and quantified using a pyruvate standard curve. The results were normalized to the protein concentration and expressed as mU/mg protein.

Hepatic AST activity was quantified with a commercial kit (E-BC-K236-M, Elabscience) in line with the manufacturer's guidelines. Liver samples were washed with PBS, homogenized, and centrifuged, and the resulting supernatants were collected for subsequent analysis. The absorbance was measured at 510 nm and quantified using a standard curve. Supernatant protein concentrations were determined via BCA assay (A65453, Thermo Scientific), and experimental results were subsequently normalized to total protein content.

### Comet assay

The assay was performed using a commercial kit (4250–050-K, R&D Systems) guided by the manufacturer's recommendations. Visualization was performed using an Olympus FV1000 confocal microscope. Image analysis was conducted with FV10 (Olympus, Shinjuku, Japan) and ImageJ software, utilizing the OpenComet plugin by which the tail moment and tail DNA (%) were calculated, indicating the extent of DNA breaks. At least four replicates were performed for each condition, with six to eight fields measured per replicate.

### Immunofluorescence

Mice were sacrificed at the indicated time points, after which their livers were collected, fixed overnight at room temperature in buffered formalin, and embedded in paraffin. The sections were then deparaffinated and rehydrated in PBS before being incubated in citrate buffer (pH 6) at 90 ºC for 30 min.

The sections were subsequently washed in PBS containing 0.05% Triton X-100 (PBS-T) and nonspecific epitopes blocked in PBS-T containing 0.2% gelatine and 2% BSA (PBS-T-G-B) for 1 h at room temperature. The sections were incubated with primary antibodies diluted in PBS-T-G-B overnight at 4 °C. The primary antibodies used are listed in Table S1.

Following being washed in PBS-T, the sections were incubated for 1–2 h at room temperature with Alexa-633-labelled secondary antibodies diluted in PBS-T-G-B. After additional washing, the sections were dehydrated and mounted in PBS:glycerol (1:1).

Hoechst 33,342 was used to stain the cell nuclei. Visualization was performed with an Olympus FV1000 confocal microscope. Fluorescence analysis was performed using FV10 software (Olympus, Shinjuku, Japan) and ImageJ software. At least four replicates were performed for each condition analysed. For each replicate, six to eight fields were measured via microscope.

Each channel was quantified independently using ImageJ/Fiji software. The images were converted to 8-bit format, and background noise was removed. To determine the numbers of total cell nuclei and marker-positive cells, automated thresholding was applied to their respective channels, followed by watershed segmentation to separate touching cells. The particles were then quantified using the “Analyse Particles” tool. The percentage of positive cells was calculated by dividing the number of marker-positive cells by the total number of nuclei in each corresponding field.

### Reverse transcription and real-time PCR

Total RNA was purified from liver tissues collected at different time points following DEN treatment. The tissue samples were mechanically homogenized, and subsequently subjected to extraction with a TRIzol reagent (Ambion)/chloroform solution, followed by centrifugation. The resulting supernatants were precipitated with isopropanol and centrifuged again at 15,000 g for 30 min at 4 °C. The pellets obtained were dissolved in DEPC-treated water and the resulting solution was further purified with a High Pure RNA Isolation Kit (Roche). Random-primed cDNA synthesis was carried out with iScript Reverse Transcription Super Mix (Bio-Rad).Reactions were carried out with Luna Master Mix (New England Biolabs) in a Step One thermal cycler (Applied Biosystems) essentially as described by [52]. Gapdh was used as the reference gene to normalize target gene expression (ΔCt), while the 2−ΔΔCt method was applied to determine changes in mRNA levels with respect to those in the untreated conditions. The sequences of the primers employed in this study are detailed in Table S2.

### Western blotting

The tissues were extracted, minced and homogenized in ice-cold lysis buffer containing 50 mM Tris–HCl (pH 7.5), 0.5% Nonidet P-40, 150 mM NaCl, 1 mM DTT, 10 mM β-glycerophosphate, 1 mM phenylmethylsulfonyl fluoride, 1 mM NaF, 1 mM sodium orthovanadate, alongside complete protease inhibitor cocktail (4 µg/µl). The supernatants obtained after centrifugation at 15,000 g for 30 min at 4 °C were used for protein quantification. Protein levels were measured with the Coomassie Plus protein assay reagent (Pierce), with bovine serum albumin used as the standard.

Aliquots containing 30 µg of total protein were electrophoresed on 5%–8%−12% or gradient SDS–PAGE gels. The separated proteins were subsequently transferred onto nitrocellulose membranes. Each membrane was cut around the molecular weight corresponding to our protein of interest and subsequently blocked in a TBS-T solution (150 mM NaCl, 50 mM Tris–HCl pH 7.5, and 0.2% Tween-20) containing 5% nonfat milk. Blots were incubated with the corresponding primary and secondary antibodies ( Table S1), washed in TBS-T and revealed using SuperSignal™ West Femto Maximum Sensitivity Substrate (Thermo Scientific).

### Immunoprecipitation

Homogenization of liver tissue was carried out in a 1 mL volume of buffer A containing 1% Triton X-100, 60 mM octyl β-D-glucopyranoside, 150 mM NaCl, 10 mM Tris–HCl (pH 7.5), 100 mM NaF, 50 mM Na3VO4, along with 1 × Complete protease inhibitor.

The were centrifuged at 15,000 g for 15 min at 4 °C, and the supernatants obtained were used to assess protein concentration. Quantification was performed with the Coomassie Plus protein assay reagent (Pierce, Rockford, IL), using bovine serum albumin as the standard. Each immunoprecipitation reaction was performed using 1 mg of protein obtained from freshly prepared extracts. The samples were precleared by incubation with 25 µl of protein A/G Plus–agarose beads for 45 min at 4 °C. Subsequently, 3 μg of anti-AhR antibody was added to the precleared samples and incubated overnight at 4 °C.

Following overnight incubation, the preparation was supplemented with 25 µl of beads and incubated for a further 45 min at 4 °C. The beads were then washed three times with Buffer B (0.15 M NaCl, 50 mM Tris–HCl, pH 7.5). Immunoprecipitation samples were analysed by Western blot or proteomics.

### Proteomics and bioinformatic/statistical analysis

Proteomic analysis was performed using liquid chromatography–tandem mass spectrometry by the Proteomics Service at the Salamanca Cancer Research Center (CSIC).

Primary data were processed using a spreadsheet. Normalization was performed by establishing the maximum signal value from the No Antibody condition as the background threshold. A subsequent binary filter was applied (1 if the signal exceeded the threshold, and 0 otherwise). Quantification of modulation was based on calculating the fold change (FC) for each condition relative to the control: FC = Experimental signal/Control signal. Filtered datasets were analysed to identify common and exclusive entities. The results of the overlap analysis were visualized using a Venn diagram generated with Venny 2.0.2 (https://bioinfogp.cnb.csic.es/tools/venny/index2.0.2.html). Gene Ontology (GO) analysis for functional annotation was performed using the DAVID platform (https://davidbioinformatics.nih.gov/). The functional groups of interest were subsequently selected from the GO results, and their behaviour across different experimental conditions was evaluated. Statistical significance for the overrepresentation of these functional groups was determined using Fisher’s exact test, yielding the associated p-values.

GraphPad Prism 6.0 software (GraphPad) was used to compare the experimental conditions. To analyse differences between two experimental groups, an unpaired two-sided Student’s t-test was used. To assess the association between two categorical variables, Fisher’s exact test was used. The data are shown as the mean ± SD. Differences were considered significant at * *p* < 0.05; ** *p* < 0.01; and *** *p* < 0.001. Data analyses are indicated in the figure legends.

## Supplementary Information

Supplementary Material 1.

## Data Availability

The authors declare that the data supporting the findings of this study are available within the paper and its Supplementary files. Should any raw data files be needed in another format they are available from the corresponding author upon reasonable request. In addition, mass spectrometry proteomics data have been deposited to the ProteomeXchange Consortium via the PRIDE partner repository [53, 54] with the dataset identifiers PXD079686 and https://doi.org/10.6019/PXD079686.
