# Supplementary material for: Aryl hydrocarbon receptor is involved in DNA damage repair after liver injury with diethylnitrosamine in mice
Source: Mol Biomed. 2026 Sep 29;7:189. doi: 10.1186/s43556-026-00596-0 (PMC13624253; doi:10.1186/s43556-026-00596-0)
Supplement: Supplementary file 1 [file 43556_2026_596_MOESM1_ESM.docx]

**MANUSCRIPT TITLE**

**Aryl hydrocarbon receptor is involved in DNA damage repair after liver injury with diethylnitrosamine in mice**

Beatriz Marín-Díaz^1,2*^, Francisco Javier González-Rico^1,2*^, Laura Ordóñez-Mata^1,2^, Ángel C. Román^1,2^, Jaime M. Merino^1,2^ , and Pedro M Fernández-Salguero ^1,2 *^

^1^ Departamento de Bioquímica y Biología Molecular y Genética, Facultad de Ciencias, Universidad de Extremadura, Badajoz 06006, Spain.

^2^ Instituto Universitario de Investigación Biosanitaria de Extremadura (INUBE) Campus de Badajoz, Avenida de la Investigación s/n, Badajoz 06006, Spain

* Corresponding authors:

Pedro M Fernández-Salguero. Email: [pmfersal@unex.es](mailto:pmfersal@unex.es)

Beatriz Marín-Díaz. Email: [beatrizmd@unex.es](mailto:beatrizmd@unex.es)

Francisco Javier González-Rico [fjgonzalez@unex.es](mailto:fjgonzalez@unex.es)

**Supplementary Fig. S1 (cleaved-caspase3).** DEN treatment induces apoptosis in both genotypes. **a** Expression of cleaved-caspase 3 analysed in both genotypes by immunohistochemistry. A NIKON TE2000U microscope was used for immunohistochemistry. Scale Bar, 50 µm. **b** Relative apoptotic cells measured as positive cell nucleus among total hepatocytes. Data are shown as mean ± SD. ∗p < 0.05; ∗∗p < 0.01; ∗∗∗p < 0.001. Groups of 3 mice were used for each genotype and time point, and measures were taken from 4-6 fields for each replicate.

**Supplementary Fig. S2** **a** Proteins identified in proteomic from AhR immunoprecipitation WT liver samples were classified according to their expression dynamics across different time points. **b** p-AKT protein levels were analysed in liver extracts by immunoblotting. Ponceau staining was used as a loading control. **c** mRNA expression of Cdk12, PolR2a, and EP300 were analysed by RT-qPCR in AhR+/+ and AhR−/− livers. Data was normalized by β-actin and is represented as 2−ΔΔCt.

| **Antibody** | **Company** | **Cat.** | **Dilution** | **Protocol** |
| --- | --- | --- | --- | --- |
| gammaH2AX | Abcam | AB81299 | 1:1000 | Western blot |
|  |  |  | 1:150 | Immunofluorescence |
| 53BP1 | Novus Biologicals | NB100304 | 1:150 | Immunofluorescence |
| AhR | Enzo Life Sciences | BML-SA210-0100 | 1:1000 | Western blot |
| AFP | Santa Cruz Biotechnology | sc-8399 | 1:200 | Western Blot |
| PCNA | Santa Cruz Biotechnology | SC-56 | 1:200 | Western Blot |
| pAKT | Cell Signaling | 9916T | 1:1000 | Western Blot |
| AKT | Cell Signaling | 9272S | 1:1000 | Western Blot |
| p38gamma | Santa Cruz Biotechnology | SC-398546 | 1:200 | Western Blot |
| Nfkβ | Santa Cruz Biotechnology | SC-8008 | 1:200 | Western Blot |
| P21 | Santa Cruz Biotechnology | SC-6246 | 1:1000 | Western Blot |
| DNA-PK | Invitrogen | MA5-32192 | 1:1000 | Western Blot |
| Bcl2 | Santa Cruz Biotechnology | SC-7382 | 1:200 | Western Blot |
| Bax | Santa Cruz Biotechnology | SC-7480 | 1:200 | Western Blot |
| Phospho-ATR | Cell Singnaling | S428 | 1:200 | Western Blot |
| Phosphor-ATM | Novus Biologicals | AF1655 | 1:1000 | Western Blot |
| Chk2 | Santa Cruz Biotechnology | SC-17747 | 1:200 | Western Blot |
| BRCA1 | Invitrogen | MA1-23164 | 1:1000 | Western Blot |
| Phosphor-BRCA1 | Cell Signaling | 9009 | 1:1000 | Western Blot |
| Cdk12 | Cell Signaling | 45791 | 1:1000 | Western Blot |
| c-casp3 | Cell Signaling | 9661S | 1:150 | Immunohistochemistry |
|  |  |  | 1:1000 | Western Blot |
| Secondary Ab (anti-Rabbit) | INVITROGEN | SA1-200 | 1:5000 | Western Blot |
| Secondary Ab (anti-mouse) | INVITROGEN | SA1-100 | 1:5000 | Western Blot |

**PRIMARY ANTIBODIES**

Supplementary Table 1.

**PRIMER SEQUENCES FOR GENE EXPRESSION ANALYSIS**

| **Name** | **Sense** | **Sequence (5´-3´)** |
| --- | --- | --- |
| Cdk12 | forward | AGTTCCGCTGACACTGATGA |
|  | reverse | CCTGGTAAAACGGAGGTCCT |
|  | | |
| BRCA1 | forward | ATCCCGGGAAAAGCTCTTCA |
|  | reverse | GGCTGCACGATCACAACTAG |
|  | | |
| EP300 | forward | TCCTGTACCTGACCCGTCTA |
|  | reverse | GTGCTGAAGAGGAGAGGGTT |
|  | | |
| PolR2A | forward | ACCGGAAGCTTACCATGGAA |
|  | reverse | CACCTCTTCCTCCTCTTGCA |

Supplementary Table 2.
